# Maternal Depressive Symptoms and Child Behavior among Mexican Women and Their Children

**DOI:** 10.3390/ijerph14121566

**Published:** 2017-12-18

**Authors:** Emily P. Flynn, Esther O. Chung, Emily J. Ozer, Lia C. H. Fernald

**Affiliations:** 1 Country Doctor Community Health Centers and Swedish Cherry Hill Family Medicine Residency, Seattle, WA 98122, USA; emilyflynn@gmail.com; 2 Division of Epidemiology, School of Public Health, University of California, Berkeley, Berkeley, CA 94720, USA; estherchung@berkeley.edu; 3 Division of Community Health Sciences, School of Public Health, University of California, Berkeley, Berkeley, CA 94720, USA; eozer@berkeley.edu

**Keywords:** maternal depression, child behavior, Mexico, indigenous health

## Abstract

Over 50% of mothers in rural Mexico have high depressive symptoms, and their children’s health and development are likely to be negatively affected. A critical question is whether children vary in their vulnerability to the effects of high maternal depressive symptoms according to their indigenous ethnicity, maternal education, or household wealth. Our sample included 4442 mothers and 5503 children from an evaluation of Mexico’s social welfare program. Maternal depressive symptoms were assessed using the Center for Epidemiologic Studies Depression (CES-D) Scale, and child behavior was measured using an adapted version of the Behavior Problems Index (BPI). Multiple linear regression models were used to explore the associations between maternal depressive symptoms and child behavior problems, and the heterogeneity of associations by indigenous ethnicity, maternal education, and household assets. We found that having greater maternal depressive symptoms was significantly associated with having a child with more behavior problems (*β* = 0.114, *p* < 0.0001, [95% CI 0.101, 0.127]), in adjusted models. In tests of heterogeneity, the association between maternal depressive symptoms and child behavior problems was strongest in households with indigenous ethnicity, low maternal education, or in households with fewer assets. These results strengthen the case for effective mental health interventions in low- and middle-income countries, particularly among the most vulnerable families where mothers and children appear to be at the greatest risk.

## 1. Introduction

Depression is common among mothers, and is a major source of disability [1,2], particularly among the poor [3]. In a study of mothers in rural Mexico, 51% were identified as having high depressive symptoms, above the US cutoff for depression [4]; similarly, a study in low-income areas of urban Mexico found that 60% of women had depressive symptoms above the US cutoff [5]. Maternal depression has been identified as a key risk factor for poor child development [6]. Given that children who grow up in poverty are already at greater risk of poor developmental outcomes, the potential effects of maternal depression on child health and development are of particular concern in low-and middle-income countries [7].

Previous research has documented the detrimental effects of maternal depression on children’s cognitive skills, motor skills, and academic achievement [8,9,10]. Maternal depression has also shown negative effects on children’s linear growth [11,12] and indicators of illness, such as diarrhea [13,14,15] and fever [15,16]. There is additional evidence that maternal depression is associated with adverse child behavioral and emotional development [17,18,19]. A recent systematic review on maternal depression and child outcomes in low-income countries highlights the risk of depression on child neurodevelopmental outcomes, but notes the lack of rigorous studies and the inconsistent findings regarding these associations [20]. In high-income countries, the risk of behavioral problems associated with maternal depression extends from early childhood to school-age and adolescence [21,22,23]. In the context of low- and middle-income countries, studies in South Africa [24,25] and Ethiopia [26] have documented associations between maternal depression and greater child behavior problems. 

Part of the explanation for these associations is that having high maternal depressive symptoms is also associated with early termination of breastfeeding [27,28,29] and reduced responsive and stimulating parenting across diverse economic and cultural groups [6,12,30]. In high-income country contexts, mothers with depression have a higher likelihood of impaired bonding with their children [31,32], poorer quality infant and child stimulation [33], and slower responsiveness to their children compared to mothers without depression [34,35]. These differences may affect children’s social-emotional development and behavior [13,36].

A question of significant policy relevance is whether there are particular subgroups of children or households within a population that are more vulnerable to the negative effects of maternal depression. If we could understand which children are more vulnerable to the effects of their mother’s depressive symptoms, it could be possible to target those mothers and children with greater support. In this paper, we explored the possible heterogeneity of associations of maternal depression and child outcomes by indigenous status, maternal education and household assets. We hypothesized that children in households with the least resources (e.g., indigenous status, low maternal education, low household assets) would be the ones more likely to show associations between maternal depressive symptoms and poor behavioral outcomes. 

## 2. Materials and Methods

### 2.1. Data Collection

Data were collected in 2003 as part of the Mexican National Social Welfare Survey (NSWS). Trained nurses from the Mexican National Institute of Public Health conducted a door-to-door survey in a random sample of 506 communities in seven Mexican states (Guerrero, Hidalgo, Michoacán, Puebla, Querétaro, San Luis Potosí, and Veracruz). All communities had fewer than 2500 inhabitants and study participants were in the poorest 20% of the Mexican population. Interviews were conducted in Spanish, with indigenous translation for a small proportion of interviews as needed. Interviewers were blinded to the aims of the study. Mothers or primary guardians (when biological mothers were unavailable) with at least one child under 6 years, or up to 72 months old, were interviewed. 

### 2.2. Sample

Mother–child pairs were included in this study if mothers were 60 years old or younger (48 mothers >60 years were excluded), children were singleton births, and the pair lived in a single-family household. Given that this survey is related to the effects of a social welfare program, and that there is evidence that participation in the program decreased maternal depressive symptoms and child behavior problems [37,38,39], we included only mother–child pairs who participated in the social welfare program; although we could have chosen only those who had not participated in the program, that sample was much smaller. With respect to missing data, mother–child pairs were included if mothers had no more than 4 of 20 (20%) depressive symptoms scale items missing and children had no child behavior scale items missing. For mothers with four or fewer depressive symptoms scale items missing, we imputed missing scores using the mean of all non-missing depressive symptoms items for each mother. 

### 2.3. Measures

#### 2.3.1. Child Behavior Problems

Child behavior was assessed using an adapted version of the Behavior Problems Index (BPI), administered via interview with the child’s mother or guardian [40]. The BPI has not been validated in Mexican rural areas; however, it was adapted to reflect items considered most appropriate for this study population and age group (Table S1). The adapted BPI has been used previously to assess child behavior in this population [37]. Scores ranged from 0 to 19 points, with a higher score indicating more behavior problems; Cronbach’s alpha for BPI was 0.81. The adapted BPI has two subscales: internalizing and externalizing. The BPI and adapted versions have been used in surveys [37,41,42,43], but it is not used clinically and does not have established clinical cutoffs. 

#### 2.3.2. Maternal Depressive Symptoms

Maternal depressive symptoms were assessed using the Spanish language version of the Center for Epidemiologic Studies Depression scale (CES-D) [44] (Table S2). The cumulative score ranges from 0 to 60 points, with a higher score indicating a greater number of symptoms; Cronbach’s alpha for the CES-D score was 0.83. Four subscales are also commonly reported: somatic, positive affect (i.e., lack of positive affect), negative affect (i.e., depressed affect), and interpersonal. The Spanish language version of the CES-D has been validated as a screening tool for depression in diverse Mexican populations [45,46]. There is some variation in CES-D scores and types of symptoms reported according to cultural and ethnic differences among Mexicans and Mexican-Americans in the US, with no equivalent increase in actual risk of clinical depression [47,48]. Therefore, appropriate clinical cutoffs are unclear for our study population. Instead of using a cutoff score for depression risk, we treated CES-D score as a continuous variable. 

#### 2.3.3. Household-Level Measures

Information was collected about number, age, and gender of household members, whether an indigenous language was spoken, level of formal educational attainment, and occupation and employment of household members. Dwelling characteristics, presence of running water and electricity, types of animals owned, and various household assets were used as indicators of household socioeconomic status (Table 1). A small number (<5%) of households had missing data; we imputed values using the community mean for those measures. To consolidate household asset information into one variable, we constructed a composite household asset index using principal components analysis for possession of eight household assets (TV, radio, gas stove, refrigerator, washing machine, blender, car, and boiler) [49]; we retained the first principal component. 

### 2.4. Statistical Analysis

We used linear, multivariate, ordinary least squares (OLS) regression and regressed each of our dependent behavior variables (total, internalizing, and externalizing child behavior scores) on each of our independent variables (total maternal CES-D score and all four CES-D subscale scores). We ran each model first unadjusted for covariates, then adjusted for child gender, mother’s age, and state of residence; a final model adjusted for these variables and all other maternal and household-level variables. Using standard techniques [50], we examined possible two-way interaction effects with total maternal CES-D score and each of the following: household ethnicity (indigenous versus non-indigenous), child’s gender, mother’s level of education (no secondary school attendance versus at least some secondary school attendance), and household asset index (5th quintile versus 1st quintile and 5th quintile versus 1st–4th quintiles). We ran each regression unadjusted and then adjusted for the same full set of covariates. For all models, we adjusted standard errors for clustering at the level of the individual mother, since there are 991 mothers who have more than one child in the sample. We conducted analyses using Stata version 14 (StataCorp., College Station, TX, USA) [51].

### 2.5. Ethical Review

This study was approved by the Research Committee of Mexico’s *Instituto Nacional de Salud Pública* and the Committee for the Protection of Human Subjects at the University of California at Berkeley. Before data were collected, study participants received a thorough explanation of the procedures and provided their informed consent to participate.

## 3. Results

There were 6964 children aged 24 to 72 months included in our original sample of participants in the social welfare program. Due to exclusion criteria, our final sample was 4442 mothers and 5503 children (Table 1); 3451 (78%) of the mothers had only one child included in the sample and 991 (22%) had more than one child. The mean maternal age was 31.08 years (SD 7.65) and the mean child age was 46.12 months (SD 13.44). Approximately 40% of the sample population was indigenous. The mean maternal CES-D score was 16.17 (0–60 point scale; SD 9.44), and 44.12% were over the US-based cutoff score of 16. The mean child behavior problems index total score was 8.22 (0–19 point scale; SD 4.20), internalizing score was 4.20 (0–10 point scale; SD 2.49), and externalizing score was 4.02 (0–9 point scale; SD 2.34). 

In all regression models (Table 2), there was a significant association between maternal CES-D score and child BPI, controlling for child age and gender, maternal age, relationship status, and education, and various household socioeconomic and demographic variables. The coefficient on total CES-D score in the fully adjusted model with total child behavior score as the dependent variable (*β* = 0.114, *p* = 0.000, [95% CI 0.101, 0.127]) represents a 0.114-point increase in behavior problems with every 1-point increase in CES-D score. This is equivalent to a 1.14-point increase in child behavior problems with a 10-point (approximately one SD) increase in maternal CES-D score. The CES-D subscales (somatic, negative affect, positive affect, and interpersonal) were also each significantly associated with total child BPI score in fully adjusted models. The associations between all combinations of CES-D subscale scores and BPI subscale scores (internalizing and externalizing) were significant.

In most models, greater child age was associated with more behavior problems, while greater maternal age, and higher asset index quintiles were associated with fewer behavior problems. In models examining total behavior score and externalizing behavior score, female gender was associated with fewer behavior problems. When examining internalizing behavior score, there were no gender differences.

**Figure 1:**
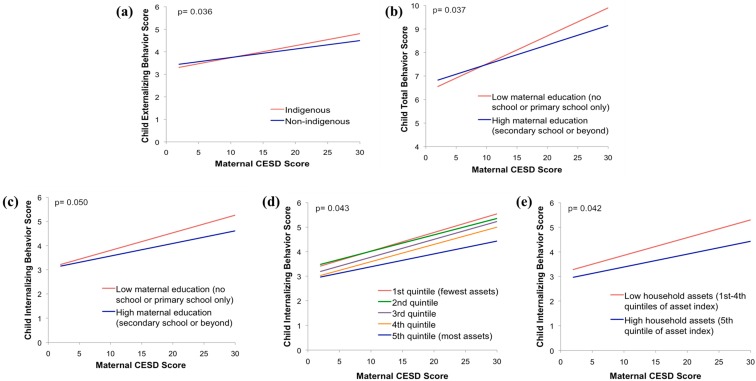
Two-way interaction effects were examined with total maternal CES-D score and (**a**) Ethnicity and child externalizing behavior score; (**b**) Maternal education and child total behavior score; (**c**) Maternal education and child internalizing behavior score; (**d**) Asset index quintiles and child internalizing behavior score; (**e**) Asset index and child internalizing behavior score. The five interactions depicted here were statistically significant at *p* ≤ 0.05 in unadjusted models.

In tests of heterogeneity, there were significant effects of indigenous ethnicity, child gender, maternal education, and household asset index (Figure 1). The strength of the association between maternal depressive symptoms and child behavior outcomes was strongest in the indigenous households (Figure 1a), in those whose mothers had the least education (Figure 1b,c), and in those coming from households with the lowest asset indices (Figure 1d,e). 

## 4. Discussion

Maternal depressive symptoms were significantly associated with greater child behavior problems. The associations between maternal depressive symptoms and child behavior problems differed significantly by indigenous ethnicity, maternal education, and household assets. The children who appeared to be the most vulnerable to the effects of their mother’s depressive symptoms were those from indigenous households, those whose mothers who had the least formal education, and those coming from households with the lowest asset indices. 

One strength of the study is that there was a large sample designed to examine rural, low-income communities in several states of Mexico. In addition, we had a high proportion of indigenous peoples included in our sample, a socially and economically marginalized group [52]. Our focus on the rural Mexican population is an essential step towards reducing health disparities and improving the health of isolated communities. 

There are limitations of this study that should be noted. First, although the literature suggests that there is a direct and unambiguous pathway from parental mental health characteristics to child development [32], our cross-sectional sample does not allow us to determine whether maternal depressive symptoms preceded or caused child behavior problems, rather than the reverse or a more cyclic causal relationship. It is theoretically possible that causality moves at least partly in the opposite direction, with child behavior problems contributing to poor maternal mental health through maternal stress or other pathways [53]. The consistent significance of the association between maternal depressive symptoms and child behavior problems in our results warrants further exploration with a study design that allows for causal inference and monitoring of maternal depression and child behavior over time. Second, the dependent variable—the adapted Behavior Problems Index (BPI)—has not been systematically validated in the rural Mexican study population, and systematic validation is needed. Third, the BPI is measured by maternal reporting and maternal depressive symptoms may influence how a mother perceives her child’s behavior problems, which could confound the relationship. Finally, the data were collected in 2003 and the findings may not reflect the true depressive symptoms in the population as this was the beginning of the economic downturn.

In the present study, maternal depressive symptoms were significantly associated with child behavior problems, across all subscales of the CES-D scale and subscales of the BPI, a finding that is consistent with other research that demonstrates adverse child behavioral development from the effects of maternal depression [18,22]. We also found increased vulnerability among children living in indigenous households, with mothers with low formal education or with fewer household assets. These findings are consistent with a recent study that found that low family income was associated with child behavior problems after adjusting for sociodemographic and maternal covariates [54]. Low-income mothers are more likely to experience depressive symptoms than their higher income counterparts [55]. Depression is associated with high indirect costs resulting in unemployment and loss of productivity [56], and the economic impact would be greater for mothers living in low and middle-income countries. Additionally, a meta-analysis of 193 publications investigating maternal depression and behavioral or positive/negative affect in children found that studies that sampled low-income families had significantly stronger effect sizes compared to those that sampled middle-income and high-income populations for externalizing behavior problems and negative and positive affect or behaviors [18]. 

Our finding that children of indigenous ethnicity were more susceptible to the effects of maternal depression on their externalizing behavior than their non-indigenous counterparts is aligned with a burgeoning body of research around the health of indigenous peoples. Geographically isolated and economically and socially marginalized, indigenous peoples experience some of the greatest health burdens and are also more likely to develop mental health disorders [57,58]. Among indigenous communities, existing poor health conditions are often heightened by exposure to numerous stressors such as racism, poverty, and unemployment [58]. A potential mechanism to explain our results is that the added effects of depression could compromise indigenous mothers’ ability to engage with their children, as has been shown in previous research in other contexts [59]. People suffering from depression are more likely to report higher levels of severe, chronic pain [60] and lower quality of life. Moreover, mothers with depression often face other challenges such as household water insecurity which can further worsen their quality of life [61]. These effects of depression can negatively affect how mothers interact with and invest in their children. Adverse conditions such as poverty may also contribute to diminished ability to develop early mother–infant attachment [24,62,63]. However, our child behavior measurement, the Behavior Problems Index, was not validated in this indigenous population and findings may not be generalizable. Still, maternal feelings of shame and guilt have been associated with depression [64,65] and depressogenic thinking and shame can lead to subsequent behavioral problems in children [66]. In previous work, maladaptive rumination on the symptoms of distress can lead to prolonged and future depressive episodes [67] and may be a promising target for both prevention and intervention strategies for mothers with depression and their children’s emotional development [68]. 

Some researchers have hypothesized potential pathways through which maternal depressive symptoms affect children’s behavioral development. For example, chronic maternal depression is associated with increased child psychopathology, and chronically depressed mothers have significantly lower levels of oxytocin compared to non-depressed mothers, suggesting that normalizing oxytocin levels may be a possible route to intervene on maternal depression and child behavioral outcomes [69]. Moreover, there may be genetic and environmental mechanisms that could explain the risk for mental health disorders in children of depressed mothers [70]. A recent study demonstrated that depressed mothers and their children had higher cortisol production than mothers with no history of psychopathology and their children. Further, cortisol production between mother and child was concordant, such that a mother’s history of depression predicted both her and her child’s cortisol production [71]. Another study examined the cortisol levels and telomere lengths of children of depressed mothers and never-depressed mothers; researchers found that children of depressed mothers had shorter telomere lengths compared to their counterparts and that shorter telomere lengths were associated with higher cortisol stress reactivity [72]. Additionally, proinflammatory cytokines such as interleukin (IL)-6 are elevated in depressed subjects [73] and positive correlations have been found between proinflammatory cytokines and antenatal depression [74]. Exposure to stressful environments, negative maternal affect and behaviors, and genetic heritability are among the potential routes through which maternal depression can lead to adverse outcomes in children [70]. 

Certain programs in low- and middle-income countries have been effective in reducing maternal depression and improving mother–child interactions, including poverty alleviation interventions [75], clinical treatments and prevention programs [76], cognitive-behavioral interventions [77], internet courses to prevent postpartum depression [78], and cost-effective smartphone applications to offer psychotherapy to mothers with depression [79]. A meta-analysis revealed that interventions in low- and middle-income countries specifically aimed at improving maternal mental health positively impacted infant development [80]. In Mexico, a group-based psychoeducational intervention facilitated by healthcare professionals was successful in reducing the incidence of clinical depression and women reported that participation in the intervention influenced their perception as a mother and improved their relationship with their infant [81]. This study demonstrates a feasible program that can be scaled up using group-based problem-solving and group social support to improve a mother’s relationship with her child. However, evidence of successful interventions among rural and indigenous communities in Mexico is scarce and further research in this area is necessary. Such interventions must be culturally adapted to the context that women live. Often, women in low- and middle-income countries reside in multigenerational households and engaging other family members to support a mother can be more beneficial than a targeted, individual approach. Including family members in providing nurturing care to an infant can also mitigate risk factors for maternal depression such as lack of personal agency and intimate partner violence [80].

## 5. Conclusions

The children in our study are among Mexico’s poorest and most vulnerable. Future research should focus on marginalized populations, particularly indigenous communities, and examine the differential vulnerability these groups face. For these children who face numerous behavioral and developmental risks, effective maternal mental health policies and programs that are culturally adapted to the indigenous context could improve individual and family mental health, development, and wellbeing.

## Figures and Tables

**Table 1:** Description of Study Participants by Variables of Interest (n = 5503 children, 4442 mothers).

Variable		Mean (SD) or %
Maternal characteristics		
	Age (years)	31.08 (7.65)
	Relationship Status	
	Married or in a relationship	93.31%
	Single, separated, widowed, or divorced	6.69%
	Education (highest level attended)	
	No school or kindergarten only	14.57%
	Some primary school	68.14%
	Some secondary school	14.66%
	Some post-secondary school	2.63%
	CES-D score ^a^ (0–60 point scale)	16.17 (9.44)
Child characteristics		
	Gender	
	Female	49.32%
	Male	50.68%
	Age (months)	48.73 (13.54)
	24–36 months	23.39%
	37–48 months	26.68%
	49–60 months	25.35%
	61–72 months	24.59%
	Behavior problems index	
	Total score (0–19 point scale)	8.22 (4.20)
	Internalizing score (0–10 point scale)	4.20 (2.49)
	Externalizing score (0–9 point scale)	4.02 (2.34)
Household characteristics		
	State of Mexico	
	Guerrero	15.04%
	Hidalgo	13.87%
	Michoacán	9.46%
	Puebla	16.50%
	Querétaro	4.21%
	San Luis Potosí	14.25%
	Veracruz	26.68%
	Indigenous ethnicity (based on language spoken)	40.21%
	Household size (number of people)	6.09 (2.28)
	Rooms in house (excluding kitchen, bathroom, passageways)	1.81 (0.99)
	Crowding (number of people per room in house)	4.13 (2.24)
	Household features	
	Land has running water	55.40%
	House has electricity	89.64%
	House has dirt floor	47.50%
	Owns draft animals (horses, mules, burros)	25.01%
	Owns grazing animals (pigs, goats, sheep)	37.66%
	Owns small animals (hens, chickens, turkeys)	57.50%
	Household assets	
	TV	65.56%
	Radio	59.48%
	Gas stove	37.75%
	Refrigerator	27.49%
	Washing machine	9.30%
	Other home appliances	54.80%
	Car	12.40%
	Boiler	3.65%

^a^ Center for Epidemiologic Studies Depression Scale.

**Table 2:** Fully adjusted models examining maternal depressive symptoms and child behavior (n = 5503 children, 4442 mothers) ^a^.

Child Behavior Outcome		*β* (95% CI)		Significant Covariates at *p* < 0.05, Variable (Direction of Effect)
Total child behavior score				
	Total CES-D score	0.114 *** (0.101, 0.127)		Child age (+), female (−), maternal age (−), asset index quintiles 3, 4, 5 (−)
	CES-D subscale scores			
	Somatic	0.298 *** (0.252, 0.344)		Child age (+), female (−), maternal age (−), asset index quintiles 4, 5 (−)
	Negative affect	0.242 *** (0.206, 0.279)		Child age (+), female (−), maternal age (−), asset index quintiles 3, 4, 5 (−)
	Positive affect	0.129 *** (0.087, 0.171)		Child age (+), female (−), maternal age (−), asset index quintiles 4, 5 (−)
	Interpersonal	0.370 *** (0.313, 0.427)		Child age (+), female (−), maternal age (−), asset index quintile 5 (−)
Internalizing child behavior score				
	Total CES-D score	0.069 *** (0.061, 0.076)		Child age (+), maternal age (−), asset index quintiles 3, 4, 5 (−)
	CES-D subscale			
	Somatic	0.176 *** (0.149, 0.202)		Child age (+), maternal advanced school (−), asset index quintiles 4, 5 (−)
	Negative affect	0.159 *** (0.138, 0.180)		Maternal age (−), maternal advanced school (−), asset index quintiles 3, 4, 5 (−)
	Positive affect	0.072 *** (0.047, 0.096)		Maternal secondary school (−), asset index quintiles 4, 5 (−)
	Interpersonal	0.228 *** (0.194, 0.262)		Asset index quintiles 4, 5 (−)
Externalizing child behavior score				
	Total CES-D score	0.045 *** (0.038, 0.052)		Child age (+), female (−), maternal age (−)
	CES-D subscale scores			
	Somatic	0.122 *** (0.097, 0.147)		Child age (+), female (−), maternal age (−)
	Negative affect	0.084 *** (0.063, 0.104)		Child age (+), female (−), maternal age (−)
	Positive affect	0.057 *** (0.035, 0.080)		Child age (+), female (−), maternal age (−)
	Interpersonal	0.142 *** (0.110, 0.175)		Child age (+), female (−), maternal age (−)

***: *p* < 0.0001. ^a^ All models included child age, child gender, maternal age, maternal relationship status, maternal education, state of residence, ethnicity, household size, and household asset index.

## Supplementary Materials

The following are available online at www.mdpi.com/1660-4601/14/12/1566/s1, Table S1: Adapted Behavior Problems Index, Table S2: Center for Epidemiologic Studies—Depression (CES-D) Scale.
