# Supplementary material for: Maternal Depressive Symptoms and Child Behavior among Mexican Women and Their Children
Source: Int J Environ Res Public Health. 2017 Dec 18;14(12):1566. doi: 10.3390/ijerph14121566 (PMC5750984; doi:10.3390/ijerph14121566)
Supplement: Supplementary file 1 [file ijerph-14-01566-s001.pdf]

Maternal depressive symptoms and child behavior in Mexican women and their children

Emily P. Flynn, MD, MPH[1], Esther O. Chung, BA[2], Emily J. Ozer, PhD[3], Lia C.H. Fernald, PhD, MBA[3*]

Table S1. Adapted Behavior Problems Index

Item in Spanish	Item in English	Internalizing subscale	Externalizing subscale
Discute mucho	Argues a lot		✓
Se queja de que se siente solo	Complains of being alone	✓	
Llora mucho	Cries a lot	✓	
Es abusador, cruel o malo con los demás	Abusive, cruel, or mean to others		✓
Exige mucha atención	Demands attention		✓
Destruye sus propias cosas	Destroys own things		✓
Destruye las pertenencias de sus familiares	Destroys family members' things		✓
Desobedece en la casa	Disobedient at home		✓
Tiene miedo de que pueda pensar o hacer algo malo	Afraid of thinking/doing something wrong	✓	
Siente que tiene que ser perfecto	Feels as though s/he has to be perfect	✓	
Siente o se queja de que nadie lo quiere	Feels as though nobody loves her/him	✓	
Siente inferior o como de no valiera nada	Feels worthless or inferior	✓	
Se involucra mucho en peleas	Fights a lot		✓
Se junta con gente que se involucra en problemas	Spends time with people who get in trouble		✓
Prefiere estar solo en vez de estar con otros	Prefers being alone to being with others	✓	
Dice mentiras o hace trampas	Tells lies, cheats		✓
Es nervioso, tenso	Is nervous, tense	✓	
Es demasiado ansioso o miedoso	Is too anxious, afraid	✓	
Se siente culpable por cualquier cosa	Feels guilty for everything	✓	

Table S2. Center for Epidemiologic Studies – Depression (CES-D) Scale

**ish**	**Item in English**	**Subscales**		
		**Somatic**	**Negative**	**Positive**
n cosas que usualmente no le molestan.	You were bothered by things that usually don't bother you.	✓		
on ganas de comer; tenía mal apetito.	You did not feel like eating; your appetite was poor.	✓		
podía quitarse de encima la tristeza aun con la amilia o amigos.	You felt that you could not shake off the blues even with help from your family or friends.		✓	
l. era tan buena como cualquier otra persona.	You felt that you were just as good as other people.			✓
ad en mantener su mente en lo que estaba	You had trouble keeping your mind on what you were doing.	✓		
rimida.	You felt depressed.		✓	
do lo que hacía era un esfuerzo.	You felt that everything you did was an effort.			
mista sobre el futuro.	You felt hopeful about the future.			✓
vida había sido un fracaso.	You thought your life had been a failure.		✓	
miedo.	You felt fearful.			
nquieto.	Your sleep was restless.	✓		
ta.	You were happy.			✓
de lo usual.	You talked less than usual.	✓		
	You felt lonely.			
ra amistosa.	People were unfriendly.			
vida.	You enjoyed life.			✓
rando.	You had crying spells.		✓	
e.	You felt sad.		✓	
le caía bien a la gente.	You felt that people disliked you.			
as de hacer nada.	You could not get "going."	✓		
